# Microstructural and Tribological Properties of a Dopamine Hydrochloride and Graphene Oxide Coating Applied to Multifilament Surgical Sutures

**DOI:** 10.3390/polym12081630

**Published:** 2020-07-22

**Authors:** Gangqiang Zhang, Jiewen Hu, Tianhui Ren, Ping Zhu

**Affiliations:** 1 College of Textile & Clothing, Institute of Functional Textiles and Advanced Materials, State Key Laboratory of Bio-Fibers and Eco-Textiles, Collaborative Innovation Center of Marine Biomass Fibers Materials and Textiles of Shandong Province, Qingdao University, Qingdao 266071, China; Hujiesen@163.com; 2 Shandong Jiejing Group, Rizhao 276800, China; 3 School of Chemistry and Chemical Engineering, Key Laboratory for Thin Film and Microfabrication of the Ministry of Education, Shanghai Jiao Tong University, Shanghai 200240, China; thren@sjtu.edu.cn

**Keywords:** surface coating, dopamine hydrochloride, graphene oxide, surgical suture, friction

## Abstract

With the development of fine surgery and desire for low-injury methods, the frictional properties of surgical sutures are one of the crucial factors that can cause damage to tissue, especially for some fragile and sensitive human tissues such as the eyeball. In this study, dopamine hydrochloride and graphene oxide were used as external application agents to prepare a biological coating for the surface of multifilament surgical sutures. The effects of this biocoating on the surface morphology, chemical properties, mechanical properties, and tribological properties of surgical sutures were studied. The friction force and the coefficient of friction of surgical sutures penetrating through a skin substitute were evaluated using a penetration friction apparatus and a linear elastic model. The tribological mechanism of the coating on the multifilament surgical sutures was investigated according to the results of the tribological test. The results showed that there were uniform dopamine and graphene oxide films on the surface of the surgical sutures, and that the fracture strength and yield stress of the coated sutures both increased. The surface wettability of the surgical sutures was improved after the coating treatment. The friction force and the coefficient of friction of the multifilament surgical sutures with the dopamine hydrochloride and graphene oxide coating changed little compared to those of the untreated multifilament surgical sutures.

## 1. Introduction

Surgical sutures are a fundamental material in surgical operation, which directly affect the results of suturing [1]. With the development of delicate surgery and desire for low-injury methods, the frictional properties of surgical sutures are one of the crucial factors that can cause damage to tissue. Multifilament surgical sutures with excellent mechanical properties and significant flexibility and pliability are crucial for suturing [2,3]. The twisted structure and the surface roughness of surgical multifilament sutures increase penetration and frictional resistance [4]. Generally, the high frictional behavior of surgical sutures is related to tissue inflammation and increases the recovery time of scars, which results in a second trauma for patients [5].

Coating is a rough surface treatment method for multifilament surgical sutures that fills the interstices between the twisted fibers and reduces the frictional resistance [6]. Various coating materials have been used to improve the frictional properties of surgical sutures; for instance, antibiotic ointment has been used to coat prophylactic surgical sutures, which decreased the coefficient of friction of the sutures when passed through tissue [6]. Antibacterial materials have been shown to reduce the maximum friction force of braided silk interacting with a skin substitute. Dopamine hydrochloride and cardiomyopathy chitosan coatings have been used to treat multifilament surgical sutures, which barely changed the coefficient of friction of the surgical sutures when sliding through a skin substitute [7].

Surgical sutures are a kind of implant material. The coating materials for sutures should be biocompatible and should barely react with tissue. Graphene oxide (GO) is widely used in the biomaterial coating field due to its superior biocompatibility and mechanical strength [8]. GO coatings have been applied to implant materials to increase their frictional performance, such as magnesium and titanium alloys [9,10]. The application of a GO coating on fibers and fabrics has also been investigated. Cai et al. [11] applied a GO coating to cotton fabric by thermal reduction under the protection of nitrogen. Chen et al. [12] grafted a GO coating onto poly(p-phenylene benzobisoxazole) (PBO) fiber by a silane coupling agent, which improved the surface roughness and wettability of the grafted fiber. Hu et al. [13] used GO, chitosan, and polyvinyl alcohol as the functional finishing agents to carry out hydrogen bond layer-by-layer self-assembly to modify the surface of cotton fabric, and the results showed that this process can form a film on the fabric’s surface. Dopamine hydrochloride (DA) is a biomaterial [14], and researchers have found that DA can be deposited onto the surfaces of various materials in a buffer solution to form a versatile platform for secondary reactions, which improves the cohesiveness and functionalization of a material [15,16,17].

The objective of this research was to prepare a DA and GO composite coating for the surface of multifilament surgical sutures and to investigate the influence of said coating on the frictional properties of the surgical sutures when penetrated through a skin substitute. The coating was characterized by mass change, a static contact angle, tensile strength, bending yield strength, and surface morphology. The impact of the coating treatment on the frictional properties of surgical sutures was investigated. The friction force of the surgical sutures was tested by using a penetration friction apparatus (PFA) [18,19], and the coefficient of friction was calculated by the elastic model and finite element simulation [20].

## 2. Materials and Methods

### 2.1. Materials

Polyglycolic acid (PGA) multifilament surgical sutures and straight stainless-steel tapered needles were purchased from Weigao Medical Instruments Co. Ltd. Sil8800 (Red, 80IRHD) artificial skin from Superior Seals has a similar toughness and constitutive function to human skin [21,22,23]. The chemical agents were of analytical grade and obtained from Aladdin Chemistry (Shanghai, China), including dopamine hydrochloride (DA), tetrahydrofuran (THF), potassium permanganate (KMnO_4_), sulfuric acid (H_2_SO_4_), sodium hydroxide (NaOH), peroxide (H_2_O_2_), phosphoric acid (H_3_PO_4_), chlorhydric acid (HCl), and absolute ethanol.

### 2.2. Synthesis and Characterization of Graphene Oxide

According to the improved Hummers method [24], GO was prepared with flake graphite as raw material, concentrated sulfuric acid as an expanding agent, and potassium permanganate as an oxidant. The preparation process was as follows: H_2_SO_4_/H_3_PO_4_ was mixed in a flask at the ratio of 9:1 (180:20 mL), graphite powder (1.5 g) was added into the continuously stirred mixture, and KMnO_4_ (9.0 g) was then slowly added. The whole mixing process was carried out in an ice bath under continuous stirring. Then, the flask was put into an oil bath and the temperature was slowly raised to 50 °C, which was then maintained for 12 h with magnetic stirring. After the reaction, the temperature of the mixture was reduced to room temperature, and 500 mL of ice was slowly added. Then, 30% H_2_O_2_ (3 mL) and 37% HCl (200 mL) were added to the lower slurry mixture, respectively. The mixture was repeatedly stirred and washed with deionized water until the pH value of the upper clear liquid was constant at 7. The microstructure, morphology, and chemical compositions were characterized by an SEM-450 (FEI Company, Hillsborough, OR, USA), AFM (Bruker, Germany), and FT-IR (Perkin Elmer, Waltham, MA, USA), respectively.

### 2.3. Coating of the Multifilament Surgical Sutures 

#### 2.3.1. The Coating Treatment

**Figure 1:**
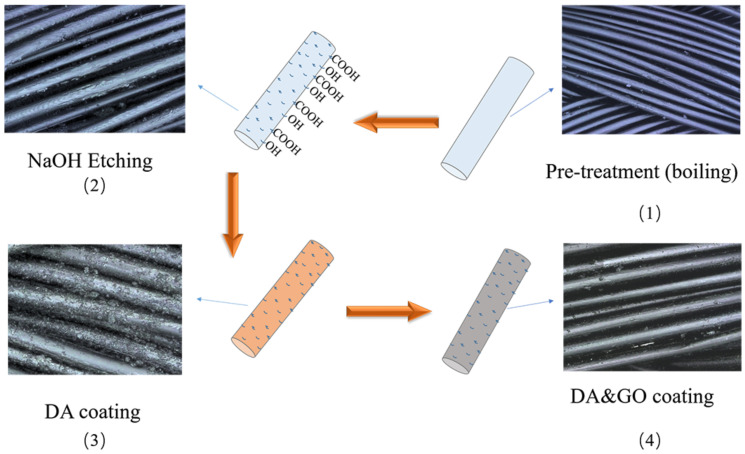
Schematic illustration of the coating process of polyglycolic acid (PGA) multifilament surgical suture. (**1**) Pre-treatment (boiling), (**2**) NaOH etching, (**3**) dopamine hydrochloride (DA) coating, (**4**) DA and graphene oxide (GO) coating.

Four steps were followed to treat the surgical sutures with the coating, namely, pre-treatment (boiling), etching, DA coating, and GO coating, as shown in Figure 1. First, the sutures’ surface coating was removed by boiling the THF solution. Second, the clear surface was etched by NaOH. Third, the etched surgical suture samples were immersed in DA Tris buffer and continuously stirred for 12 h at room temperature. Finally, the surgical sutures were coated with the GO slurry by the dip-coating method.

#### 2.3.2. Surface Analysis

A laser scanning microscope (VK 9700, KEYENCE, Osaka, Japan) was used to evaluate the topography and three-dimensional (3D) profiles of the sutures. FT-IR (Perkin Elmer, USA) was used to characterize the chemical composite of the surface of the surgical sutures.

#### 2.3.3. Mechanical Properties

##### Weight Change

The weight change (*w*) of the treated surgical sutures was calculated by Equation (1):

(1) $$w=\frac{w_{2}-w_{1}}{w_{1}}\times 100\;\%$$

where *w*_1_ is the weight of the untreated sutures and *w*_2_ is the weight of the treated sutures.

##### Contact Angle

Water contact angle measurements (Data Physics OCA20, Germany) were used to characterize the hydrophilicity of the surface of the sutures by the sessile drop method at room temperature [25]. A camera recorded images of 3 μL of water dropped onto tight sutures. The contact angle was measured by the OCA20 software. The five measurements were repeated.

##### Tensile Strength Test

A tensile tester (Zwick/Roell 500N, Germany) was used to measure the strength of the sutures. The samples were cut into lengths of 25 cm with a moving velocity of 10 mm/min until the sutures broke. The tensile strength and the elongation at the break of the sutures were recorded by a computer [26].

##### Bending Stiffness

Bending stiffness is an important parameter that influences the friction properties of surgical sutures [27]. In this study, the cantilever method was used to estimate the bending stiffness of the surgical sutures. The load was applied to 1 cm parts of the sutures, with a total length of 5 cm. Then, 15 s later, the distance at which the end of the surgical sutures falls under the load and is placed on the horizontal plane was measured [28]. Next, 1 cm-long sutures were suspended in the air, and the bending stiffness was computed according to Equation (2).

(2) $$B=\frac{F*l^{3}}{3f}$$

where *B* is the bending stiffness, *l* is 1 cm in this study, *f* is the deflection of the sutures’ loading end, and *F* is the dead load.

### 2.4. Tribological Measurement

**Figure 2:**
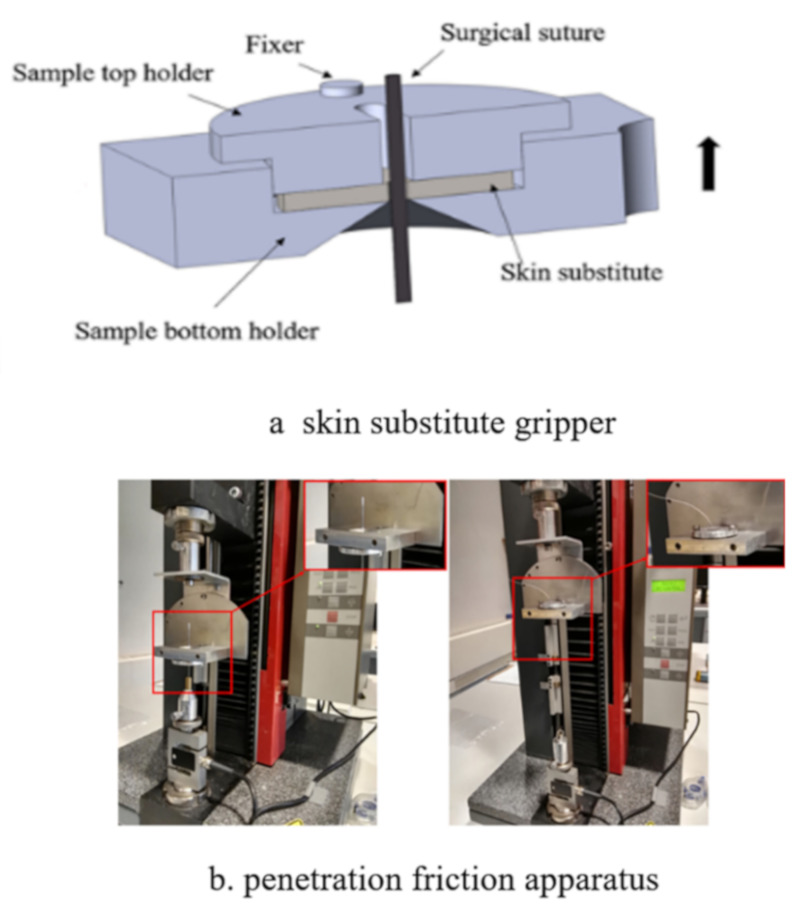
Penetration friction apparatus (PFA): (**a**) Skin substitute gripper; (**b**) penetration friction apparatus.

The friction and wear properties of the surgical sutures using artificial skin were tested by a PFA [20], as shown in Figure 2. The specific operation was as follows: The sample fixture was assembled on a Zwick/Roell tensile strength tester (500 N, Germany). When the surgical sutures penetrated through the friction tester, the friction force of the surgical sutures passing through the tissues or organs was evaluated. In the friction measurement, the artificial skin was fixed in the gripper. Then, one end of a surgical suture was left free, and the other penetrated the skin using the surgical suture needle and was fixed onto the force measuring sensor. The experiment was repeated three times for each experimental parameter, and the average value of the experimental results was taken. Table 1 shows the experimental parameter conditions.

## 3. Results and Discussion

### 3.1. Characterization of Graphene Oxide

**Figure 3:**
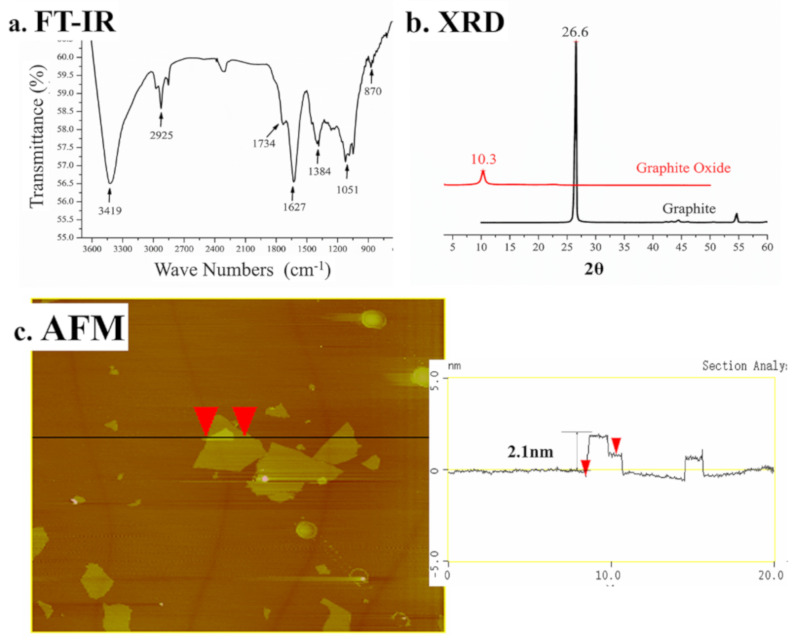
Characterization of GO: (**a**) FT-IR, (**b**) XRD, and (**c**) AFM tapping mode image and height profile.

The chemical components of GO were evaluated by FT-IR. From Figure 3a, in the spectrum, the O–H groups of GO at 3419 cm^−1^ can be observed. The stretching vibrations of the C=O and C=C of GO were 1734 and 1627 cm^−1^, respectively. The C–O vibrations in C–OH and the C–O–C vibrations in epoxy were observed at 1384 and 1051 cm^−1^, respectively. The interlayer spacing of graphite and GO was assessed by XRD according to Bragg’s law, as shown in Equation (3).

(3) nλ=2dsinθ

From Figure 3b, it can be seen that the reflection of GO is a single peak at 2*θ* = 10.3°, illustrating that the layer spacing is larger. Due to the oxide groups in GO, the water molecules were trapped between the graphene oxide sheets [29,30]. No obvious peak was found in the profile of GO, indicating that the graphite was successfully oxidized to GO. From Figure 3c, it can be seen that the thickness of the GO sheet was 0.93 nm and that the stacking of the GO sheet was 2.1 nm, which is in accordance with the values for the single-layer GO sheet [31]. 

### 3.2. Surface Chemical Composite and Morphology 

**Figure 4:**
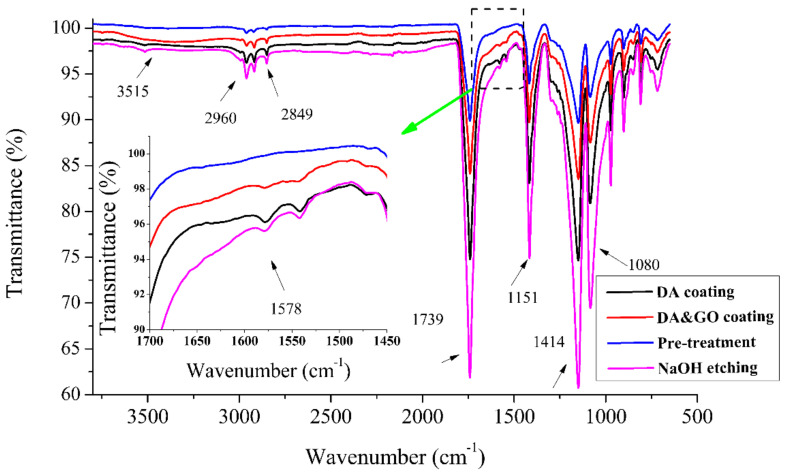
FT-IR of the polyglycolic acid (PGA) multifilament surgical sutures with and without different treatments (i.e., NaOH treatment and DA/GO coatings). The plot inside is a magnification of the infrared spectrum.

The surface chemical composites of the PGA surgical sutures with different treatments were confirmed by FT-IR. From Figure 4, the stretching vibration of –OH with a carboxyl group (–C=O–OH) can be found in the FT-IR spectrum at 3515 cm^−1^. The bending vibration peaks of C=O in carboxyl (–C=O–OH) were at 1080 and 1414 cm^−1^, and the stretching vibration peaks of C=O in carboxyl (–C=O–OH) was observed at 1739 cm^−1^. The characteristic absorption peak on the surface of the surgical sutures etched by NaOH was stronger. Therefore, it can be concluded that more carboxyl groups were produced after NaOH treatment. It can also be seen from the figure that the stretching vibration absorption peak of –NH/–OH appeared between 3600 and 3100 cm^−1^ for the PGA multifilament surgical sutures after the DA and GO coating treatment. The bending characteristic absorption peak of –NH was 1578 cm^−1^, and the stretching characteristic absorption peaks of C–O were 1151 and 1080 cm^−1^ [32,33,34]. This confirms that the DA and GO coating adhered to the surface of the surgical sutures.

**Figure 5:**
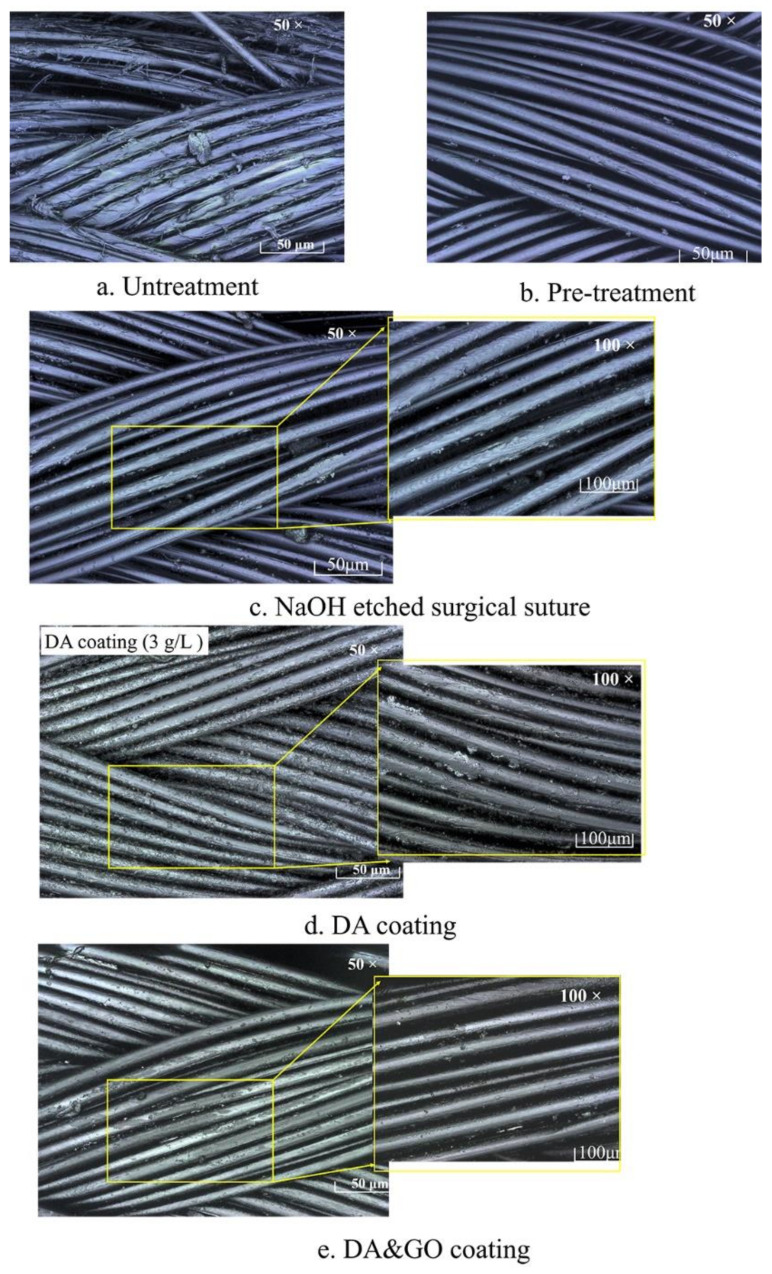
Morphology of the surface of the surgical sutures with different treatments. (**a**) Untreated suture; (**b**) THF treatment; (**c**) NaOH-etched suture; (**d**) DA coating; (**e**) DA and GO coating.

Figure 5 shows the surface morphology of the surgical sutures with and without a coating treatment. After the THF treatment, the coating material was removed from the surface of the commercialized surgical sutures. After NaOH etching, the small cracks and dents on the surface of the surgical sutures and fibers became thinner. The defects and specific surface area of the surface of the fiber became larger, providing more adhesion points for the subsequent coating. 

After the DA coating, more DA coating particles accumulated on the surface of the sutures. The small DA particles in the buffer solution deposited onto the surface of the fiber to form a uniform film [14], so the adhesion to the surface of the sutures was enhanced. The DA coating provided good reaction conditions for the subsequent coating treatment as a secondary reaction platform [15,16,17]. After the GO coating, a uniform GO film formed on the surface of the suture, which became smoother, and the gap between the fibers was filled.

### 3.3. Mechanical Properties

**Figure 6:**
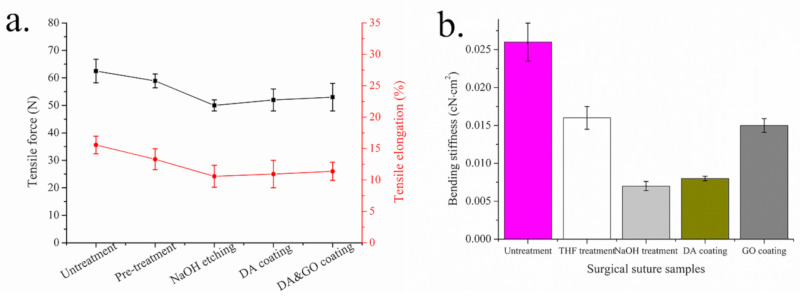
Tensile force, tensile elongation, and bending stiffness of the PGA multifilament surgical sutures. (**a**) Tensile force and elongation; (**b**) bending stiffness.

Tensile fracture strength is an important parameter of surgical sutures. If the tensile breaking strength is too low, the sutures are easily pulled and slipped when they pass through tissues, leading to knots. Too low a tensile fracture strength shortens the absorption time of sutures after an operation, resulting in unsatisfactory suturing and increasing the risk of operation failure. Figure 6a shows the tensile strength and elongation at break of the sutures left untreated and those with NaOH etching and a DA and GO coating. The error bars in the graphs indicate the standard deviation of each measurement. It can be seen that when the sutures were treated with NaOH, the tensile strength of the PGA multifilament surgical sutures decreased from 58.9 to 50.0 N and the elongation at break from 30.4% to 24.2%. This may be because the loss of surgical sutures with the NaOH etching destroyed the structure of the fiber and created defects. It can also be seen from the figure that the increasing range of the tensile strength and elongation at break of the surgical sutures after the DA and GO coating treatment is limited. This is because the adsorption of the coating on the surgical suture is limited, and the adsorption capacity of the coating material cannot continue to increase during the coating treatment.

The bending yield property of surgical sutures is an important parameter that affects the friction between surgical sutures and tissues. From Figure 6a, it can be seen that the bending yield strength of the commercial surgical sutures without any treatment was 0.026 cN·cm^2^, which reduced to 0.016 cN·cm^2^ after removing the protective layer by THF treatment, and 0.007 cN·cm^2^ after NaOH treatment. After the DA coating, the suture bending yield strength increased from 0.007 to 0.008 cN·cm^2^. Then, with GO coating treatment, the bending yield strength of the suture increased to 0.015 cN·cm^2^. These results are similar to previous research results [7,28]. 

The surfaces of the surgical sutures currently on the market are covered by a coating. If the coating is removed, the fibers of multifilament surgical sutures with a multifilament braided structure have no adhesion for the coating, the cohesion between the fibers is reduced, and the bending yield strength is reduced more. NaOH treatment further reduced the strength and diameter of the fibers, and, at the same time, the cohesion between the fibers was further decreased and the surgical sutures were able to bend more easily. However, after the DA coating treatment, the bond strength of the fiber was weak, and so an increase in the bending yield strength was not obvious. For the surgical sutures treated with GO, because GO can form a film on the surface of surgical sutures with DA particles, it filled the gaps between the fibers of the surgical sutures, which improved the cohesion between the fibers, increased the overall hardness of the surgical sutures, and improved the bending yield strength.

**Figure 7:**
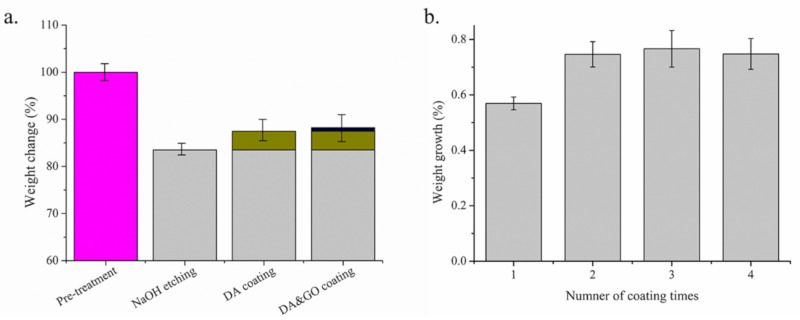
Weight change of the surgical sutures. (**a**) Weight change of the multifilament surgical sutures treated with different treatments; (**b**) weight change of the multifilament surgical sutures with a GO coating.

Mass change rate is an important parameter to evaluate the effects of treatment on surgical sutures. Figure 7a shows the mass change rate of the surgical sutures with and without a coating treatment. It can be seen that after NaOH etching, the mass loss rate of the PGA multifilament surgical sutures was 16.5%. When the surgical sutures were coated with DA, the mass increase rate was 3.92%. After three GO coating treatments, the mass increase rate of the PGA multifilament sutures was 0.75%.

Figure 7b shows the effect of the GO coating treatment on the mass change rate of the surgical sutures with and without a coating. It can be seen that with the increase in the number of coating treatments, the weight of the PGA multifilament surgical suture increases. After three coating treatments, the increase in the mass change rate of the PGA multifilament sutures tended to stabilize. In this experiment, the PGA sutures were treated with three GO coatings.

### 3.4. Wettability Properties

**Figure 8:**
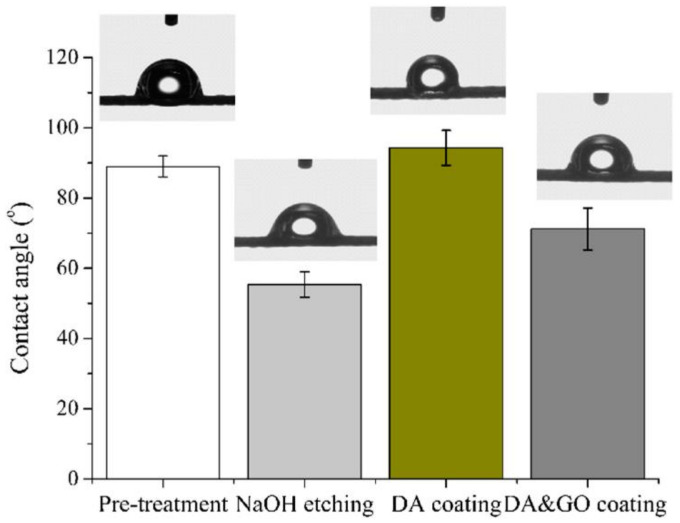
Water contact angle of the multifilament surgical sutures with different treatments.

NaOH etching and coating of multifilament surgical sutures influence the wettability properties of the surface. Figure 8 shows the static water contact angle of the surgical sutures with and without a coating treatment. After NaOH etching, the static contact angle of the surface of the sutures decreased to 58°. This is due to the fact that the hydrolysis reaction on the surface of the sutures increased the number of –OH and –COOH groups, which improved their wettability. After the DA and GO coating treatments, the static water contact angle of the surgical sutures increased to 91°and 72°, respectively.

### 3.5. Tribological Properties

#### 3.5.1. Frictional Properties

**Figure 9:**
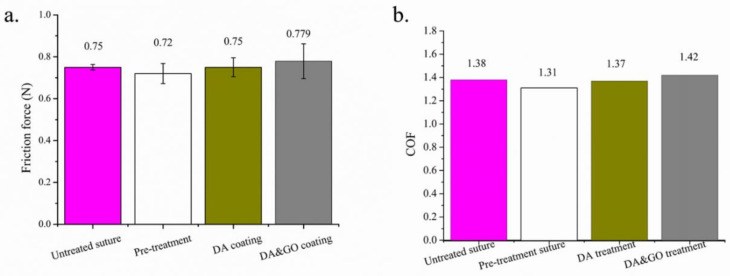
Friction properties of the PGA multifilament surgical sutures with DA and GO coatings. (**a**) The friction force; (**b**) the coefficient of friction.

Figure 9a shows the impact of the coating treatment on the friction force of the multifilament surgical sutures. The friction force of the sutures with THF, DA, and GO coating treatments was 0.72, 0.75, and 0.779, respectively. The friction force differed little between those sutures that did or did not receive a coating treatment.

The coefficient of friction (COF) is a curricular impact factor that assesses tribological properties, which are defined as the friction force and the normal force. The friction force was tested by PFA, while the normal force of the surgical sutures penetrating through the artificial skin was predicted by a simplified linear elastic model, which is based on the Hertz contact model [18,35].

(4) $$F_{N}=2EhL\frac{r_{suture}}{a}\int_{0}^{a}\sqrt{\frac{a^{2}-x^{2}}{R^{2}-x^{2}}\;}dx\;$$

According to the classical frictional equation (Equation (5)):

(5) $$\mu =F_{f}/F_{N}$$

The thickness of the etching and coating was much less than the diameter of the surgical sutures in this contact condition. From Figure 9b, it can be seen that the COF of the surgical sutures without treatment, with DA, and with GO was 1.38, 1.37, and 1.42, respectively.

#### 3.5.2. Wear Properties

**Figure 10:**
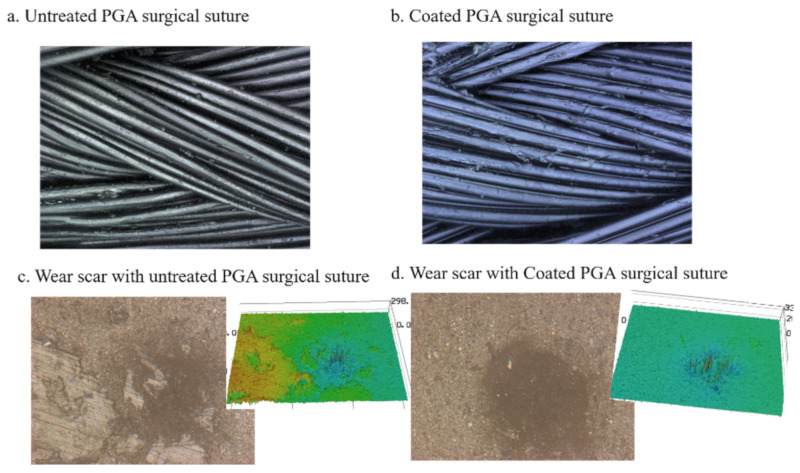
Wear morphology of the surgical sutures and the skin substitute. (**a**) Untreated surgical suture; (**b**) coated surgical suture; (**c**) wear scar with untreated surgical sutures; (**d**) wear scar in skin substitute with coated surgical sutures.

The wear morphologies of the surgical sutures and skin substitutes are shown in Figure 10. It can be seen that there is little wear debris present on the commercialized and coated surface of the sutures, as shown in Figure 10a,b. There is little difference between the coated surgical sutures and the commercialized surgical sutures. In order to further study the wear performance of the surgical sutures with the DA and GO coating, we observed the three-dimensional wear morphology of the surface of the artificial skin. The wear crack in the skin substitute was similar to that of the commercialized and coated sutures, as shown in Figure 10c,d.

#### 3.5.3. Mechanism

In this study, the surgical sutures with different treatments—including commercial suture and sutures with either a DA coating or a DA and GO coating—presented similar friction and wear properties. This may be explained by the fact that there was little change in the high stiffness and roughness of sutures after the coating treatment. Moreover, the experimental condition in this paper was dry friction.

In the contact model, the friction force was composited with adhesion and the deformation component [36].

(6) $$F_{f}=F_{f,adh}+F_{f,def}$$

The adhesion component was the main component of the friction force due to the surgical sutures and the skin substitute being viscoelastic materials. The adhesion component was determined by the product of the shear strength τ and the real contact area $A_{r}$ between them [37]:

(7) $$F_{f,adh}=\tau A_{r}$$

**Figure 11:**
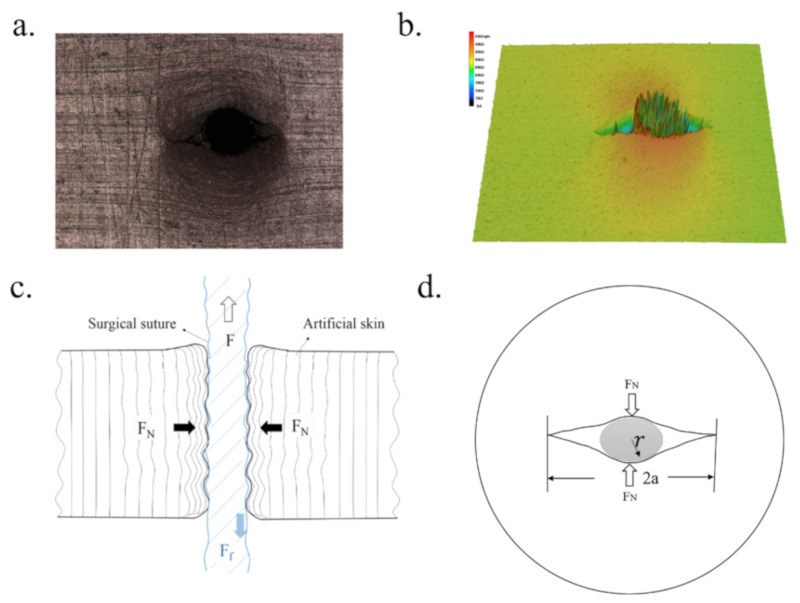
(**a**) Image of a surgical suture penetrating the skin substitute; (**b**) three-dimensional (3D) image of the surgical suture inserted into the skin substitute; (**c**) sketch of the surgical suture penetrating through the skin substitute; (**d**) force analysis of the surgical suture.

The shear strength of the surgical sutures and the skin substitute was identified. The contact area was the most effective factor in the frictional properties of the surgical sutures. To better reveal the influence of the GO coating on the tribological mechanism of the skin substitute, the artificial skin was penetrated with a surgical suture, as shown in Figure 11a,b. It can be seen that when the surgical suture was inserted into the planar crack in the skin substitute, the surgical suture deformed under the pressure. Furthermore, the cohesion force of the twisted fibers was low. The resilience force of the skin substitute acted symmetrically on the two surfaces of the surgical suture, as shown in Figure 11c,d. Hence, the contact area was similar between surgical sutures with and without a coating, which presented similar frictional properties.

## 4. Conclusions

In this study, a DA and GO coating was applied to the surface of multifilament sutures to form a uniform film. The COF of the PGA multifilament sutures with and without a DA and GO coating was 1.37 and 1.42, respectively, which were in the same range. According to the contact mode and the way in which the surgical sutures are inserted, the contact surface was the main factor affecting the friction properties of the surgical sutures penetrating through the skin substitute. Due to the twist structure of surgical sutures, the contact area was similar when the surgical sutures were inserted into the skin substitute; hence, the coating barely affected the frictional properties of the surgical sutures.

## Figures and Tables

**Table 1:** Experimental parameters.

Test-Related Factors	Instructions
Equipment	Penetration friction apparatus
The diameter of the space of gripper	25 ± 0.5 mm
Puncture angle	90°
Puncture velocity of the needle	60 mm/min
Puncture distance of the needle	10 mm
Penetration velocity of the suture	100 mm/min
Penetration distance of the suture	100 mm
